# Factors associated with discontinuation of treatment for pulmonary arterial hypertension in the United States

**DOI:** 10.1002/pul2.12326

**Published:** 2024-04-15

**Authors:** Harrison W. Farber, Hayley D. Germack, Nicole S. Croteau, Jason C. Simeone, Fei Tang, Carly J. Paoli, Gurinderpal Doad, Sumeet Panjabi, Teresa De Marco

**Affiliations:** ^1^ Division of Pulmonary, Critical Care and Sleep Medicine Tufts Medical Center Boston Massachusetts USA; ^2^ Janssen Scientific Affairs, LLC Titusville New Jersey USA; ^3^ Real World Evidence Cytel Waltham Massachusetts USA; ^4^ Actelion Pharmaceuticals Titusville New Jersey USA; ^5^ Division of Cardiology University of California, San Francisco San Francisco California USA

**Keywords:** pulmonary arterial hypertension, risk factors, treatment

## Abstract

Information on factors leading to pulmonary arterial hypertension (PAH) treatment discontinuation is limited. This study analyzed 12,902 new PAH medication users to identify predictors of treatment discontinuation. Treatment by accredited pulmonary hypertension centers and combination therapy with PAH agents from different classes were less likely to result in discontinuation.

## INTRODUCTION

Pulmonary arterial hypertension (PAH) is a progressive disease characterized by elevated pulmonary arterial pressure, leading to right ventricular failure and death. Therapies for three pathophysiologic targets are currently available to manage PAH. Persistence, or time on continuous PAH treatment, is crucial for disease management and improved outcomes.^1^

Discontinuation rates can vary widely among PAH patients. A 2022 systematic literature review and meta‐analysis of seven studies found the proportion of patients discontinuing disease‐specific PAH therapies varied from 27% to 73%.^1^ Factors associated with discontinuation are poorly understood. This study aimed to investigate treatment discontinuation among patients with PAH and identify predictors of discontinuation by therapeutic class.

## METHODS

This retrospective observational study analyzed US medical and pharmacy claims data (Komodo Healthcare Map™) from January 2015 to May 2022. The database included 330 million patients across the United States. Study eligibility requirements included age ≥18, initiating PAH treatment with a phosphodiesterase (type 5) enzyme inhibitor (PDE5i), endothelin receptor antagonist (ERA), soluble guanylate cyclase (sGC) stimulator, or prostacyclin pathway agent (PPA) between January 2016 and November 2021 with 12 or more months baseline medical and drug plan enrollment and at least one inpatient or two outpatient pulmonary hypertension (PH)/PAH claims in the baseline period (international classification of diseases, ninth revision (ICD‐9‐CM) 416.0, 416.8; ICD‐10‐CM I27.0, I27.20, I27.21, I27.89). Treatment initiation served as the index date. PPAs were subdivided into parenteral (intravenous, subcutaneous, inhaled) and oral subclasses. For patients taking multiple medication classes on index, PPAs parenteral, and oral were classified as the highest priority, followed by ERA, sGC stimulators, and PDE5i.

Exclusion criteria included PAH treatment, chronic thromboembolic pulmonary hypertension diagnosis or treatment, lung transplant, or atrial septostomy during the baseline period (12 months before index date). Only PAH‐approved PDE5i doses were considered, with a minimum of 1 pill/day to exclude patients treated for erectile dysfunction. Patients were followed until the earliest of disenrollment, death, or end of study period.

Baseline characteristics and treatment class‐level persistence were assessed. Treatment episodes were calculated using days' supply from pharmacy claims and start and end dates for clinically administered medications; early medication refills were appended to the end of the previous fill's supply. Drug switches within a therapeutic class were considered treatment continuations. Patients were assumed to continue therapy during inpatient stays. Discontinuation was defined as treatment gaps ≥ 60 days.

Proportions of patients who discontinued treatment, median persistence from Kaplan–Meier analysis, and effect estimates of predictors of discontinuation from multivariable Cox proportional hazard models for each therapeutic class were reported. Eligible covariates included class‐specific combination therapy defined as the use of two or more PAH classes at index, services from a Pulmonary Hypertension Association accredited care center (PH CC)^2^ at or before index therapy, baseline demographics, and clinical characteristics. The identification of patients seen at a PH CC involved a multistep process. First, the National Plan and Provider Enumeration System National Provider Identifier (NPI) registry was searched for accredited centers using a list of PH CC sourced from the Pulmonary Hypertension Association. Komodo provided a list of anonymized healthcare organization identifiers (HCO ID) associated with the primary healthcare organization (HCO) linked to these NPIs. Patients were classified as seen at a PH CC if they received care from any provider associated with the HCO list provided. Stepwise models were adjusted for payer type and other covariates with *p* < 0.01 in univariable analyses; the PPA oral model was also adjusted for combination therapy. Adjusted hazard ratios (HR) and corresponding 95% confidence intervals (CI) were reported.

## RESULTS

In total, 12,902 patients met the study criteria. The average age was 60.9 years and 60.9% were female. The most common comorbidities included hypertension (81.1%), dyspnea (68.2%), congestive heart failure (CHF; 65.7%), diabetes mellitus (38.7%), coronary artery disease (38.3%), renal disease (35.5%), and atrial fibrillation (AF; 27.6%). Most patients were classified as PDE5i users (76.1%), followed by ERA (11.4%), parenteral PPA (8.3%), sGC stimulators (3.0%), and PPA oral (1.2%). Combination therapy use on index date was rare: 0.3% of sGC stimulator patients, 3.2% parenteral PPA, 15.0% ERA, and 16.8% oral PPA; 45% of patients attended a PH CC at baseline. Mean number of oral drugs for all classes (PAH and other drugs) on index was 6.4 (standard deviation: 3.9) with a median of 6 (interquartile range: 3–9) and mean number of pills (PAH and other drugs) on index was 11.8 (standard deviation: 8.2) with a median of 10 (interquartile range: 6–16).

Discontinuation was most common among parenteral PPA users (65.8%; mean follow‐up 20.5 months), followed by PDE5i (60.4%; 20.9 months), sGC stimulators (56.2%; 25.6 months), oral PPA (52.8%; 21.4 months), and ERA (49.2%; 24.5 months). Similarly, parenteral PPA users had the shortest median persistence in months (6.5, 95% CI: 5.6‐7.9), followed by PDE5i (7.7, 95% CI: 7.1–8.1), oral PPA (15.1, 95% CI: 6.6–28.8), sGC stimulators (16.1, 95% CI: 11.9–21.6), and ERA (22.9, 95% CI: 20.4–26.9). In sensitivity analyses, median overall persistence to any PAH drug class was higher than persistence to index class as follows: index class parenteral PPA (11.2 months), PDE5i (8.9 months), oral PPA (40.1 months), sGC stimulators (20.0 months), and ERA (32.5 months).

**Figure 1:**
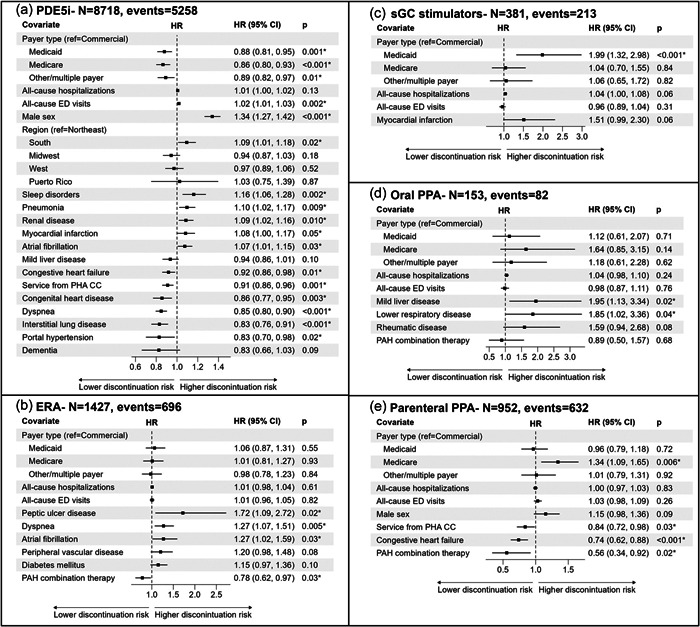
Cox models for discontinuation by therapeutic class: (a) PDE5i, (b) ERA, (c) sGC stimulators, (d) oral PPA, and (e) parenteral PPA. *p < 0.05. CI, confidence interval; ED, emergency department; ERA, endothelin receptor antagonist; HR, hazard ratio; PAH, pulmonary arterial hypertension; PDE5i, phosphodiesterase (type 5) enzyme inhibitor; PHA CC, Pulmonary Hypertension Association accredited care center; PPA, prostacyclin pathway agent; sGC, soluble guanylate cyclase.

Factors associated with discontinuation of PAH treatment are presented in Figure 1 stratified by therapeutic class. Statistically significant factors (*p* < 0.05) associated with higher risk of discontinuation for PDE5is included male sex (HR = 1.34), sleep disorders (HR = 1.16), pneumonia (HR = 1.10), Southern US census region (HR = 1.09), renal disease (HR = 1.09), myocardial infarction (MI; HR = 1.08), AF (HR = 1.07), and increased baseline emergency department visits (HR = 1.02). Factors associated with a lower risk of discontinuation included portal hypertension (HR = 0.83), interstitial lung disease (HR = 0.83), dyspnea (HR = 0.85), congenital heart disease (HR = 0.86), noncommercial insurance plans (Medicare HR = 0.86; Medicaid HR = 0.88; or other/multiple payers HR = 0.89), care from PH CC (HR = 0.91), or CHF (HR = 0.92).

Statistically significant factors associated with higher hazard of ERA discontinuation included peptic ulcer disease (HR = 1.72), dyspnea (HR = 1.27), and AF (HR = 1.27). Receiving ≥2 PAH therapeutic classes on the index was associated with a lower hazard of discontinuation (HR = 0.78). Among sGC stimulator users, Medicaid insurance (HR = 1.99) was associated with an increased discontinuation hazard. Mild liver disease (HR = 1.95) and lower respiratory disease (HR = 1.85) were associated with an increased discontinuation hazard among oral PPA users.

No factors significantly decreased discontinuation hazard for sGC stimulator or oral PPA users. For parenteral PPA users, Medicare insurance was associated with an increased hazard of discontinuation (HR = 1.34). However, receiving ≥2 PAH therapeutic classes on index (HR = 0.56), CHF (HR = 0.74), and care from a PH CC (HR = 0.84) was associated with a decreased hazard of discontinuation.

## DISCUSSION

This US retrospective observational study investigated treatment discontinuation among new users of PAH therapies and identified class‐specific drivers of discontinuation. Results indicate that PDE5is were most used, followed by ERAs, parenteral PPAs, sGC stimulators, and oral PPAs, consistent with other published studies.^3^, ^4^, ^5^, ^6^ Discontinuation varied by therapeutic classes and ranged from 49.2% (ERA) to 65.8% (parenteral PPA) over mean follow‐ups of 20.5–25.6 months, which corroborates previous studies.^4^ Discontinuation rates were particularly high among patients using parenteral PPAs (65.8% discontinued), possibly due to switching to oral PPAs, inappropriate drug administration at initiation, or side effects. While this study did not investigate reasons for discontinuation, other studies have show PAH treatment complexity, side effects, cost, and inadequate social support to be common reasons for discontinuation. These studies suggest adopting simplified treatment regimens, early identification and management of side effects, financial assistance programs, and patient education as important factors for PAH treatment adherence and persistence.^1^, ^3^, ^4^, ^5^, ^6^

The comorbidity burden of included patients indicates that a significant proportion may meet criteria for World Health Organization (WHO) group 2 (PH due to left‐sided heart disease) and 3 (PH due to lung disease). Therefore, findings are less specific for PAH patients, but may be generalizable to PH patients (WHO groups 1–5). However, this study provides valuable insights into the complexities and challenges of managing therapies for PAH/PH in routine clinical practice.

Several factors were linked to a lower risk of discontinuation. Care from a PH CC was associated with a decreased hazard of discontinuation by 16% for parenteral PPA and 9% for PDE5i users. PAH treatment initiation with class‐specific combination therapy was associated with a lower discontinuation hazard by 44% for parenteral PPA users and 22% for ERA users compared to initiation with monotherapy.

Male PDE5i users were more prone to treatment discontinuation. While efforts were made to include only those receiving PAH treatment, patients primarily treated for erectile dysfunction may have remained in the analyses.

Several comorbidities were associated with an increased risk of discontinuation including AF (PDE5i, ERA); sleep disorders, pneumonia, renal disease, and MI (PDE5i); peptic ulcer disease and dyspnea (ERA); and mild liver disease and lower respiratory disease (oral PPA). Interestingly, other comorbidities such as CHF (PDE5i, parenteral PPA) and portal hypertension, interstitial lung disease, dyspnea, congenital heart disease (PDE5i) were associated with a lower risk of discontinuation. Increased contact with healthcare providers managing these comorbidities may contribute to better monitoring and management of PAH. In general, in this study patients were older with multiple comorbidities. Expert centers are better equipped to manage such complex patients; however, many patients are not receiving care from PH CCs (54.9%), presenting an opportunity for collaboration between expert and nonexpert centers.

Initial inappropriate treatment with PAH‐specific therapies may contribute to treatment discontinuation and suboptimal outcomes. The present study identified a reduced discontinuation hazard among patients treated at PH CCs which may indicate benefit of expertise in PAH diagnosis and management.

Several limitations should be noted. The imprecision of administrative claims data may result in misclassification of study variables or missing data, which can affect the validity and generalizability of the findings. Specifically, no PAH‐specific ICD‐10 codes currently exist. Second, sample sizes of sGC and oral PPA groups were relatively small, and caution should be exercised when interpreting these model results. Claims indicate medication dispensation only.

These results provide insights into several aspects PAH patient care: (1) use of PAH medications; (2) factors associated with discontinuation of these medications; and (3) lower discontinuation rates at PH CCs. In sum, they suggest that healthcare providers should consider these issues when treatment is initiated and monitored. Moreover, they provide another reason why PAH patients should be seen/followed at PH CCs: such expertise, especially in educating, supporting, and managing these patients is essential to medication compliance. Further study and eventual mitigation of these issues are warranted to improve the outcomes of PAH patients.

## CONFLICTS OF INTEREST STATEMENT

Hayley D. Germack, Sumeet Panjabi, and Carly Worden were employees of Janssen Scientific Affairs, LLC at the time of this study and Gurinderpal Doad is an employee of Janssen Pharmaceuticals and may own stocks. Nicole Croteau, Jason Simeone, and Fei Tang are employees of Cytel, which has received consultancy fees from Janssen Scientific Affairs, LLC for the conduct of this study. Harrison Farber and Teresa DeMarco provided consulting services to Janssen Scientific Affairs, LLC. Harrison Farber has received speaking honoraria from Bayer and SAB fees from Acceleron (Merck), Actelion (Janssen), Altavant, Aerami, Aerovate, and United Therapeutics. Teresa De Marco is a United Therapeutics advisory board member and has received consulting fees from Aerovate, Actelion/Janssen/Johnson & Johnson, Boston Scientific, Natera, NXT, Pulnovo, Scope‐Bial, and Tectonic.

## ETHICS STATEMENT

As the study used only deidentified patient data that was already available, it was exempt from ethical or institutional review board review/approval.
